# The Treatment Outcomes of Olfactory Neuroblastoma Patients With Frontal Lobe Invasion

**DOI:** 10.3389/fonc.2021.640892

**Published:** 2021-07-05

**Authors:** Jie Wang, Li Wang, Huanyu He, Yi Li, Xinmao Song

**Affiliations:** ^1^ Department of Radiation Oncology, Eye, Ear, Nose & Throat Hospital of Fudan University, Shanghai, China; ^2^ Department of Oncology, 920th Hospital of Joint Logistics Support Force, Kunming, China

**Keywords:** olfactory neuroblastoma, frontal lobe invasion, endoscopic resection, radiotherapy, prognosis

## Abstract

**Background:**

To investigate whether frontal lobe invasion (FLI) was an unfavorable prognostic factor in patients with olfactory neuroblastoma (ONB), and to explore the optimal treatment strategy for ONB patients with FLI.

**Methods:**

Some 37 patients with FLI were retrospectively studied, and 74 well-matched patients without FLI were enrolled as the control group. The long-term survivals were compared between the two groups.

**Results:**

No significant differences were found between the two groups in overall survival (OS), progression-free survival (PFS), locoregional failure-free survival (LRFS), and distant metastasis-free survival (DMFS) (all p >0.05). Multivariate analyses showed that FLI wasn’t an independent predictor for OS (HR = 1.100, 95% CI = 0.437–2.772, p = 0.840). Among the 37 patients with FLI, patients who received surgery combined with chemo-/radiotherapy showed better OS (89.4% *vs.* 53.6%, p = 0.001) and PFS (87.8% *vs.* 53.6%, p = 0.001) compared with those who didn’t undergo surgery.

**Conclusions:**

FLI wasn’t a poor prognostic factor for ONB patients. Endoscopic resection combined with radiotherapy was an effective therapeutic method for ONB patients with FLI.

## Introduction

Olfactory neuroblastoma (ONB), which was first described in 1924 by Berge et al., is a rare neuroendocrine tumor that derives from the olfactory neuroepithelium (1). While its etiology remains unclear, it accounts for approximately 3% of nasal cavity and sinus tumors (2). ONB may occur in children or adults, and the incidence rate peaks at the age of 60–80 (3). There is no distribution disparity found between males and females.

ONB exhibits variable biological behaviors ranging from indolent growth to highly aggressive proliferation with the potential for regional and distant metastases. The symptoms in the early phrases are non-specific, with the most common symptoms being nasal obstruction and epistaxis, which are difficult to arouse people’s attention. Consequently, most patients present with locally advanced disease at diagnosis. Even though the tumor arises from the olfactory cells, anosmia is not a common complaint (4). Because the olfactory area has a direct extension to the central nervous system through the thin-walled cribriform plate, the neoplasm could easily erode the anterior skull base and invade into the frontal lobe. Once tumor extends to the frontal lobe, patients typically develop associated symptoms including headache, nausea, epilepsy, and even psychiatric symptoms, which makes treatment difficult.

On the basis of current evidence, surgical resection combined with radiotherapy with or without chemotherapy is the primary treatment modality for the localized tumor. This regimen yields promising survival rates and local control rates (5, 6). Cervical lymph node metastasis, positive surgical margins, and advanced Kadish stage have been shown to be the independent factors associated with poor prognosis in patients with ONB (3). Given the rarity of ONB, no studies have been conducted to evaluate the treatment and outcomes of patients with tumor invasion into the frontal lobe. The intended purpose of this study is to discuss whether frontal lobe invasion (FLI) is an unfavorable prognostic factor for patients with ONB and to investigate the clinical outcomes of comprehensive treatment in ONB patients with FLI. Moreover, the optimal treatment strategy for ONB with FLI is also delineated.

## Material and Methods

Thirty-seven patients of ONB with FLI were enrolled in this study. Furthermore, according to the FLI to no-brain-involvement incidence ratio of 1:2, an additional 74 ONB patients without FLI were selected as the control group. Sex, age, staging, and treatment modalities were well-matched between the two groups. All of the ONB patients were confirmed by pathology, and were treated and followed at the Eye, Ear, Nose and Throat Hospital of Fudan University between July 1991 and October 2018. The study was approved by the hospital’s institutional review board, and the informed consent was waived because of the retrospective nature of the study. All patients underwent enhanced computed tomography (CT) and/or magnetic resonance imaging (MRI) scan of the head and neck before treatment. Brain invasion was assessed by the enhanced MRI and/or CT of head and neck. All of the 37 patients with frontal lobe invasion showed brain tissue invasion as well as edema of the surrounding brain tissue on MRI and/or CT. Patients with distant metastasis were excluded in this study.

The long-term survival outcomes, including overall survival (OS), progression-free survival (PFS), locoregional failure-free survival (LRFS), and distant metastasis-free survival (DMFS) were evaluated. Pearson chi-square test was utilized for categorical variables. Survival analyses were performed using the Kaplan–Meier method. The log-rank test was used for comparison of survival between two groups. The Cox proportional hazards model was used to identify independent prognostic factors for OS. P-value was 2-tailed and considered statistically significant when less than 0.05. Statistical analyses were performed with SPSS version 20.0 (IBM Corp., Armonk, NY, USA).

## Results

In our study, 37 patients with frontal lobe invasion were recruited, and an additional 74 well-paired patients without intracranial invasion were also included as the control group. There was no statistically significant difference between the two groups in terms of clinical characteristics and treatment methods (**Table 1**). Among the 37 patients with FLI, those who received surgery combined with postoperative radiotherapy were confirmed by the surgeons to have brain invasion; those who received radical radiation therapy or preoperative radiotherapy had significantly shrinkage in tumor size after radiotherapy, suggesting brain invasion. According to the Hyams grading, among the 37 cases, there were three cases of grade I, six cases of grade II, 22 cases of grade III, and six cases of grade IV. About 24.3% of tumors were classified as low-grade (Hyams I/II), and 75.7% of tumors were with high grade (Hyams III/IV).

**Table 1:** Patients’ clinical characteristics, n = 111.

	FLI (−), n = 74	FLI (+), n = 37	χ^2^	p
	n (%)	n (%)		
Age				
<60	54 (48.6)	28 (24.3)	0.093	0.760
>60	20 (18.0)	9 (8.1)		
Gender				
Male	54 (48.6)	29 (26.1)	2.287	0.130
Female	20 (18.0)	8 (7.2)		
Kadish stage				
C	59 (53.2)	29 (26.1)	0.027	0.868
D	15 (13.5)	8 (7.2)		
T stage^a^				
T1–T2	13 (17.6)	0 (0)	7.362	0.004
T3–T4	61 (82.4)	37 (100)		
N stage^a^				
N negative	59 (53.2)	29 (26.1)	0.027	0.868
N positive	15 (13.5)	8 (7.2)		
IR technique				
2D-RT	3 (2.7)	2 (1.8)	0.014	0.907
3D-CRT	39 (35.1)	19 (17.1)		
IMRT	32 (28.8)	16 (14.4)		
Treatment modality				
IR plus S	61 (55.0)	30 (27.0)	0.030	0.861
IR	13 (11.7)	7 (6.3)		
Chemotherapy				
No	14 (12.6)	5 (4.5)	0.508	0.476
Yes	60 (54.1)	32 (28.8)		

FLI, frontal lobe invasion; IR, irradiation therapy; S, surgery.

^a^According to the Dulguerov–Calcaterra staging system.

**Figure 1:**
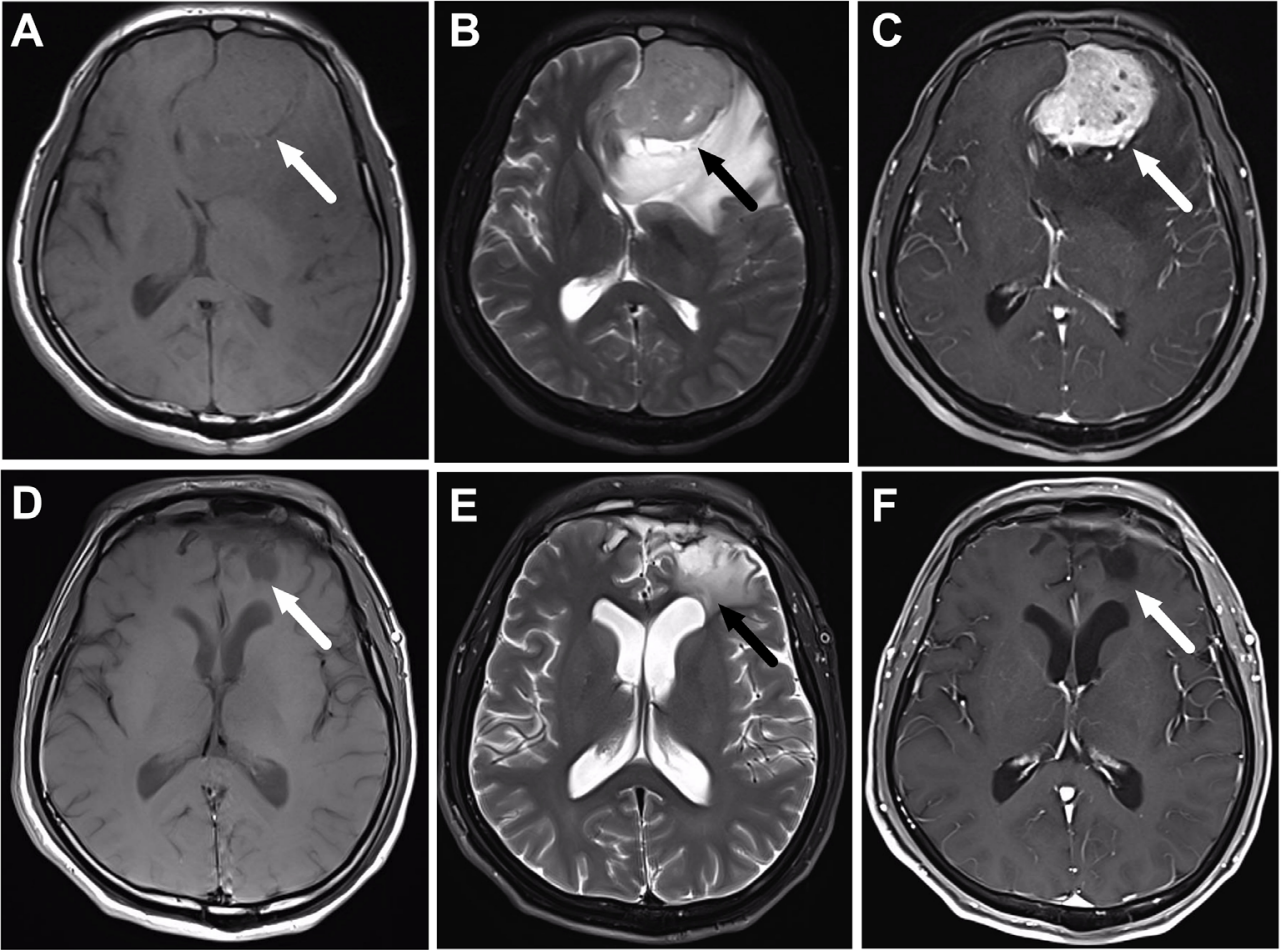
A 56-year-old male patient with olfactory neuroblastoma of Kadish Stage C underwent surgical resection followed by chemoradiotherapy. **(A–C)** are the magnetic resonance (MR) images before treatment, while **(D–F)** are the corresponding MR images after the completion of treatment. **(A)** Axial T1-weighted MR imaging reveals an isointense mass in the frontal lobe (arrow). The mass has moderate signal intensity on T2-weighted MR images **(B)**, and shows heterogeneous enhancement on the post-contrast T1-weighted MR images **(C)**. **(D–F)** show that the tumor totally disappears after the completion of treatment, and there is still edema of the frontal lobe (arrow).

Among the 37 patients with FLI, 29 patients were male and the other eight were female. The age at diagnosis ranged from 26 to 76 years, with a median age of 48 years. The symptoms at initial diagnosis included nasal obstruction (59.5%), epistaxis (59.5%), dysosmia (24.3%), exophthalmos (15.6%), palpable neck masses (9.4%), visual changes (6.3%), and eye pain (3.1%). According to the modified Kadish staging system, 29 patients were with Kadish stage C, and the other eight patients were with stage D. The tumors showed hypointense signal on T1-weighted images and moderate to high signal intensity on T2-weighted images; the tumors showed heterogeneous enhancement on post-contrast T1-weighted images. The tumors consistently displayed an intact envelope, with the adjacent brain tissues and ventricles compressed, deformed, or even displaced. Cerebral edema was found in almost every patient with frontal lobe invasion (**Figure 1**).

Treatment modalities in the 37 patients with FLI included surgery, radiotherapy, and chemotherapy. Radical radiation therapy and/or chemotherapy without surgery were performed in seven patients (18.9%). Of these patients, some refused surgical treatment, while others either could not tolerate surgery due to poor overall condition or had involvement of skull base and/or orbit making it difficult to achieve complete tumor excision. The other 30 patients underwent surgical resection combined with chemo-/radiotherapy, including 12 cases having preoperative radiotherapy and 18 cases having postoperative radiotherapy. There were 12 patients with orbital invasion or massive FLI, and preoperative radiotherapy was performed to decrease the tumor burden, which could reduce the scope of surgery, and protect the critical structures as much as possible. The surgical procedures performed included endoscopic-assisted transcranial surgery (21/37, 56.8%), lateral rhinotomy (5/37, 13.5%), open craniofacial resection (3/37, 8.1%), and craniofacial resection combined with endoscopic surgery (1/37, 2.7%).

Radiotherapy (RT) was performed daily with 180–225 cGy per fraction in all of the 37 patients, and the mean dose to the tumor bed was 6470 cGy (range, 5,480–7,200 cGy). About 51.4% (19/37) patients underwent three-dimensional conformal RT (3D-CRT), 43.2% (16/37) patients had intensity-modulated RT (IMRT), and only 5.4% (2/37) patients were treated with two-dimensional conventional RT (2D-RT). Elective neck irradiation was performed for all the 37 patients with FLI. Bilateral regions of VIIa and II were irradiated in patients without cervical lymph node metastasis. In patients with cervical lymph node metastasis, the ipsilateral regions of VIIa, Ib, II;, III, IV, and V were irradiated.

Among the 37 patients with FLI, 32 patients were treated with chemotherapy in a 21-day cycle. Some 18 patients had induction chemotherapy, 4underwent induction chemotherapy followed by concurrent chemotherapy, five had induction + concurrent + adjuvant chemotherapy, two were given with induction + adjuvant chemotherapy, two had concurrent chemotherapy, and one was treated with concurrent + adjuvant chemotherapy (**Supplementary Table 1**). The primary drugs were vincristine, epirubicin, pirarubicin, cyclophosphamide, and cisplatin in the induction and adjuvant chemotherapy settings, and cisplatin was used in the concurrent chemotherapy setting.

After completing the comprehensive treatment, 36 patients achieved complete or near-complete response, and only one patient showed stable disease. Complete response was defined as the disappearance of the lesions and the short axis of any pathological lymph nodes being less than 10 mm after treatment; near-complete response was defined as there being at least a 70% decrease in the longest diameter of lesion, taking the longest diameter before treatment as baseline. After operation, there were one case of encephalic infection and four cases of cerebrospinal fluid leakage. The chemotherapy and radiation toxicities were recorded. Acute grade 3 radiodermatitis occurred in three cases, but no acute grade 4 or 5 dermatitis was found. Four patients had grade 3/4 leukopenia. Six patients developed acute conjunctivitis. All patients had different degrees of nasal dryness. About 48.6% (18/37) of patients had local frontal lobe edema. About 62.2% (23/37) of patients had olfactory dysfunction. No mental disorder or epilepsy occurred.

**Figure 2:**
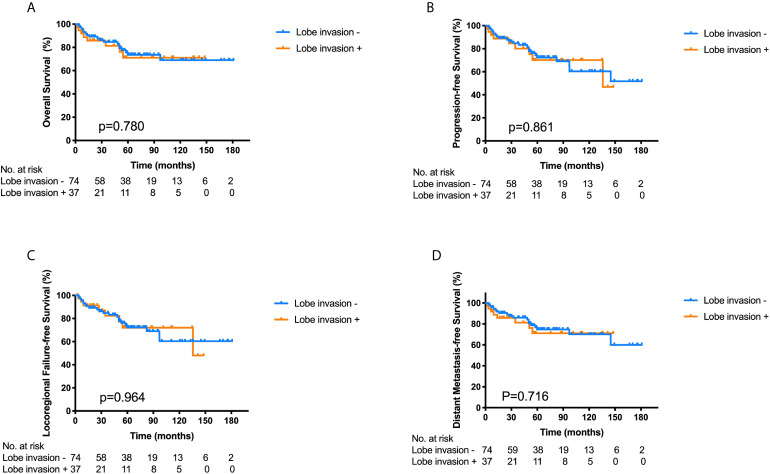
Kaplan–Meier survival curves for overall survival **(A)**, progression-free survival **(B)**, locoregional failure-free survival **(C)** and distant metastasis-free survival **(D)** of the ONB patients with frontal lobe invasion (FLI) (n = 37) and those without FLI (n = 74), p-values calculated by log-rank test.

The median follow-up time was 34.5 months (range, 2.5–148 months) in the 37 patients. Seven patients (18.9%) died, two patients (5.4%) had a local relapse, one patent (2.7%) had a regional failure, and one patient (2.7%) developed distant metastasis to the bone. The 3- and 5-year OS, PFS, LRFS and DMFS for the 37 patients with FLI were 81.3%/71.1%, 80.1%/70.1%, 82.4%/72.1%, and 81.3%/71.1%, respectively. On the other hand, the 3- and 5-year OS, PFS, LRFS and DMFS for the 74 patients without FLI were 84.7%/73.7%, 84.7%/72.3%, 84.7%/72.3%, and 85.9%/74.7%, respectively. There were no significant differences in the 3-year OS (p = 0.780), PFS (p = 0.861), LRFS (p = 0.964) and DMFS (p = 0.716) between the two groups (**Figure 2**). Hence, FLI wasn’t a poor prognostic factor for ONB patients.

**Figure 3:**
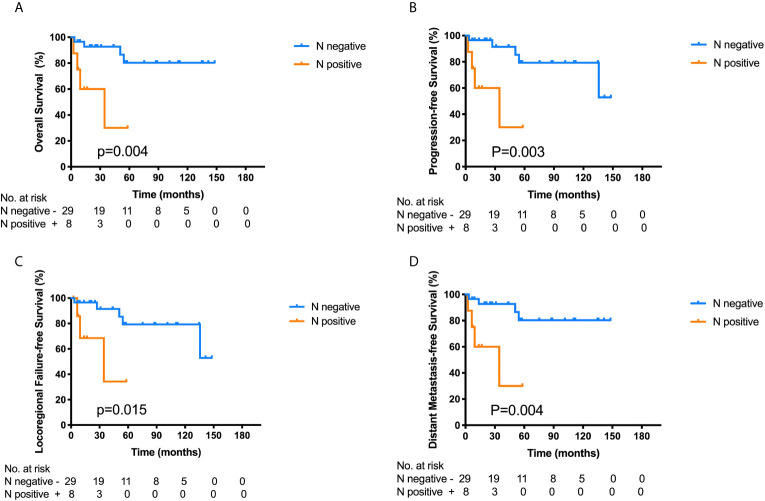
Kaplan–Meier survival curves for overall survival (OS) **(A)**, progression-free survival (PFS) **(B)**, locoregional failure-free survival (LRFS) **(C)** and distant metastasis-free survival (DMFS) **(D)** of the 37 ONB patients with frontal lobe invasion according to the cervical lymph node metastasis at initial diagnosis. Log-rank tests show that ONB patients without lymph node metastasis have superior 3-year OS (p = 0.004), PFS (p = 0.003), LRFS (p = 0.015), and DMFS (p = 0.004) compared with those having cervical lymph node metastasis.

Among the 37 ONB patients with FLI, the Log-rank analysis showed that there were significant differences in the 3-year OS (30.0% *vs.* 92.7%, p = 0.004), PFS (30.0% *vs.* 91.5%, p = 0.003), LRFS (34.3% *vs.* 91.5%, p = 0.015) and DMFS (30.0% *vs.* 92.7%, p = 0.004) between patients with cervical lymph node metastasis (LNM) at initial diagnosis and those without cervical LNM (**Figure 3**). Additionally, the multivariate analyses also identified that cervical lymph node metastasis was significantly correlated with worse OS in all the 111 patients (**Table 2**). Hence, cervical lymph node metastasis was an independent predictor of poor prognosis in ONB patients.

**Table 2:** Univariate and multivariate analyses of factors in relation to overall survival using the cox proportional hazards model (n = 111).

Variables	Univariate analyses			Multivariate analyses		
	HR	95% CI	p-value	HR	95% CI	p-value
Age (>50 *vs.* <50)	0.715	0.291–1.755	0.427	0.833	0.345–2.012	0.685
Frontal lobe invasion (No *vs.* Yes)	0.888	0.379–2.085	0.780	1.100	0.437–2.772	0.840
Cervical LN (N0 *vs.* N+)	0.309	0.109–0.877	0.002	0.231	0.094–0.567	0.001
Surgery (No *vs.* Yes)	0.202	0.072–0.5637	0.000	0.215	0.092–0.503	0.000
Chemotherapy (No *vs.* Yes)	1.304	0.487–3.495	0.567	1.298	0.453–3.717	0.627
Orbital invasion (No *vs.* Yes)	0.440	0.202–0.960	0.035	0.586	0.244–1.406	0.231

**Figure 4:**
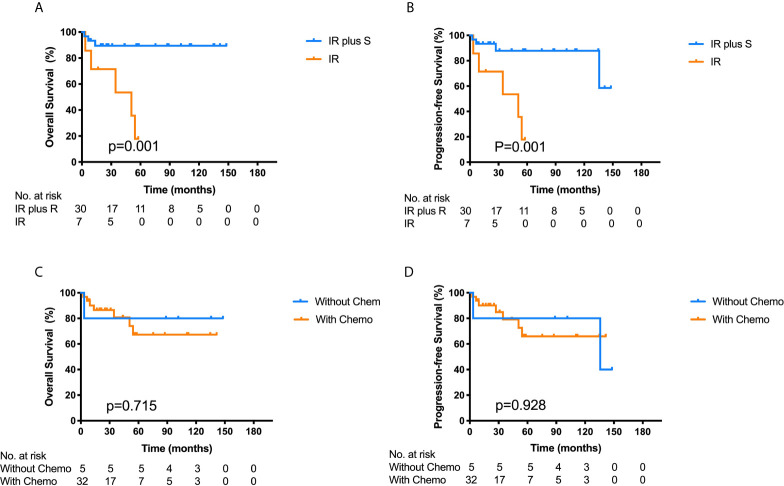
Kaplan–Meier survival curves for overall survival (OS) **(A)** and progression-free survival (PFS) **(B)** of the 37 ONB patients with frontal lobe invasion stratified by IR plus S *vs.* IR. Log-rank tests show that the combination of IR and S has better OS (p = 0.001) and PFS (p = 0.001) than IR alone. Kaplan–Meier survival curves for OS **(C)** and PFS **(D)** of the 37 ONB patients according to chemotherapy (without Chemo *vs.* with Chemo). Log-rank test shows no significant differences between the two groups. IR, irradiation therapy; S, surgery; Chemo, chemotherapy.

Among the 37 ONB patients with FLI, patients who received surgery combined with chemo-/radiotherapy showed significantly better OS (89.4% *vs.* 53.6%, p = 0.001) and PFS (87.8% *vs.* 53.6%, p = 0.001) than those who did not undergo surgery (**Figures 4A, B**). Among the 21 patients who underwent endoscopic approaches, only one patient died of an uncontrolled lesion, and the others had no locoregional failure or distant metastasis. The 3-year OS in patients receiving endoscopic resection combined with chemo-/radiotherapy was 95.2%, slightly higher than that of 76.2% in patients treated with other surgical approaches, and the 3-year PFS was 76.2 and 88.9% in the two groups respectively. Hence, endoscopic resection combined with radiotherapy was an effective therapeutic method for ONB patients with FLI.

Among the 37 ONB patients with FLI, 32 patients had undergone chemotherapy, while the other five patients were without chemotherapy. Although most cases were sensitive to chemotherapy, the 3-year OS and PFS for patients treated with chemotherapy was 80.8 and 79.2% respectively, while the patients who did not receive chemotherapy had a 3-year OS and PFS of 80.0% respectively. The log-rank analysis showed that there were no significant differences in the 3-year OS and PFS between the two groups (p = 0.715, 0.928) (**Figures 4C, D**). Furthermore, for patients receiving chemotherapy, the 5-year OS and PFS rates were 60.4 and 64.1% in patients with high-grade tumors (Hyams III and IV), and 83.3 and 72.9% in patients with low-grade tumors (Hyams I and II). There was no significant difference in 5-year OS (P = 0.203) and PFS (P = 0.560) between the two groups using log-rank test. Multivariate analysis showed that Hyams grade had no significant effect on 5-year OS (95% CI: 0.449–12.792, P = 0.306) and PFS (95% CI: 0.352–7.459, P = 0.551), as well as chemotherapy on OS (95% CI: 0.069–15.074, P = 0.988) and PFS (95% CI: 0.153–16.504, P = 0.699) (**Supplementary Table 2**). Hence, chemotherapy may not play a vital role in the treatment of ONB with FLI.

## Discussion

There is currently no consensus on the long-term outcomes and optimal treatment regimen for ONB patients with frontal lobe invasion (FLI) due to its rarity. In this study, we retrospectively studied the clinical records of 37 patients who had tumor invasion to the frontal lobe from a single institution, and investigated the optimal treatment strategy. To the best of our knowledge, this is the largest cohort of ONB patients with FLI, which has never been reported before.

Dulguerov et al. reported that the 5-year OS and disease-free survival rates were 45 and 41% in patients with esthesioneuroblastoma, and surgery combined with radiation therapy achieved the best average survival rate of 65% (7). Another meta-analysis showed that the 5-year OS stratified by therapy modality was 73% for surgery combined with radiotherapy, 68% for surgery only, 35% for radiotherapy only, and 26% for neither surgery nor radiotherapy, suggesting that the combination of surgery and radiotherapy was the optimal treatment for patients with ONB (8). However, little is known about the treatment strategies and outcomes for ONB patients with FLI. The major concerns in clinical practice include the selection of craniotomy or endoscopic surgery, the radiotherapy details, and the exact role of chemotherapy. In our study, the 3- and 5-year OS, PFS, LRFS and DMFS for patients with FLI were 81.3%/71.1%, 80.1%/70.1%, 82.4%/72.1%, and 81.3%/71.1% respectively, which were not inferior to those of the 74 well-matched patients without FLI. Multivariate analysis also showed that frontal lobe invasion was not an independent predictor of poor prognosis for ONB patients (**Table 2**) (HR = 1.100, 95% CI = 0.437–2.772, p = 0.840). Therefore, brain tissue invasion might not be a challenge to prognosis if the optimal therapeutic strategy was selected.

Surgery played an extremely important role in the multidisciplinary treatment of ONB with FLI. In the 37 patients with FLI, patients who received surgery combined with chemo-/radiotherapy showed better OS (89.4% *vs.* 53.6%, p = 0.001) and PFS (87.8% *vs.* 53.6%, p = 0.001) than those who did not undergo surgery (**Figures 4A, B**). The most common treatment modality (21/37, 56.8%) was endoscopic resection combined with chemo-/radiotherapy. Hence, surgery in combination with chemo-/radiotherapy may be the optimal treatment mode for ONB patients with FLI. Craniotomy was thought to be the optimal surgical modality for the treatment of ONB patients with intracranial involvement. However, craniotomy may lead to complications such as cerebrospinal fluid leak, meningitis, brain abscess, mental status changes, hematoma, and hemorrhage (9–11). While craniotomy can achieve adequate access to the invaded portion of the anterior skull base in advanced ONB, the transnasal approach should be used in selective patients on a case-by-case basis with a careful review of imaging and patient’s anatomy (12). The surgeons will evaluate the feasibility of complete resection of tumor under endoscope before operation. If the tumor can be completely removed, endoscopic surgery will be chosen due to the less damage and better postoperative skull base repair. In addition, the relationship between the tumor and intracranial vessels should be evaluated. If the tumor is close to vessels, endoscopic surgery will increase the risk of bleeding, then the craniotomy is needed as hemostasis is difficult under endoscope.

With advances in endoscopic techniques, transnasal endoscopic approaches could achieve an en bloc resection with minimal facial scarring and less complications. Endoscopic resection of skull base tumors showed superior neurological, visual, and functional outcomes, as well as better recovery compared with open approaches (13). A meta-analysis of 26 studies containing 609 patients revealed that the endoscopic approaches had comparable control rates to the craniofacial resection in the treatment of ONB (14). A multicenter retrospective study on stage-matched ONB patients reported the favored survival in the endoscopic surgery group with Kadish C stage compared with the open resection group; however, no significant differences were found in survival between the two groups with Kadish B stage (13). Among the 37 ONB patients with FLI, 21 patients underwent endoscopic resection combined with chemo-/radiotherapy, and none showed any serious complications. The 3-year OS in patients receiving endoscopic resection combined with chemo-/radiotherapy was 95.2%, which was higher than that of 76.2% in patients who accepted other surgical approaches. Hence, endoscopic resection combined with chemo-/radiotherapy may be an effective therapeutic method for ONB patients with FLI.

Increasing studies have presented the value of chemotherapy in the treatment of ONB, and it is recommended as an acceptable strategy for aggressive and locally advanced disease (15, 16). It is reported that ONB is sensitive to chemotherapy in patients who have induction chemotherapy, and the chemosensitivity of ONB depends on its biological characteristics (17). The application of chemotherapy may be considered in patients with advanced stage, high Hyams grade, extensive regional disease, distant metastases, positive margins, unresectable tumors, and recurrent tumors (18). The chemotherapy regimens vary at different institutions, and no consensus exists regarding the chemotherapy modality. Platinum-based neoadjuvant chemotherapy, which consists of etoposide, ifosfamide, and cisplatin, is effective in the treatment of ONB (19). However, Kiyota et al. reported that nonplatinum-based induction chemotherapy followed by definitive radiotherapy was also a promising option for locally advanced diseases (20). In our study, 86.5% (32/37) of the patients received platinum-based chemotherapy, and 78.4% (29/37) of them received neoadjuvant chemotherapy. Although some patients presented tumor regression after induction chemotherapy, no benefits of OS and PFS were achieved in patients having chemotherapy compared with those without chemotherapy. According to the Hyams grade system, chemotherapy showed slightly better OS and PFS in low-grade tumors than those in high-grade tumors, and there was no statistical significance. On multivariate analysis, chemotherapy and Hyams grade had no significant influence on OS and PFS.

Since the particle beam radiation therapy (PBRT) could enhance the therapeutic ratio compared with photon-based intensity-modulated radiotherapy (IMRT) due to its particular dosimetry characteristics, it has been reported to have great result in in the treatment of head and neck neoplasms. Up until now, there are very few reports on the utilization of PBRT for the treatment of ONB, and no study showed the comparison between the therapeutic effect of PBRT and photon-based IMRT. A retrospective study of 12 ONB patients treated with PBRT reported a 2-year OS, PFS, LRFS, and DMFS rates of 83.3, 75.8, 87.5, and 79.5%, respectively (21). In our study, all the 111 patients were treated with photon-based CRT or IMRT. The 3-year OS, PFS, LRFS and DMFS for the 37 patients with FLI were 81.3, 80.1, 82.4, and 81.3%, respectively. And the 3-year OS, PFS, LRFS and DMFS for the 74 patients without FLI were 84.7, 84.7, 84.7, and 85.9%, respectively. Our results were not inferior to those using PBRT. Hence, a prospective study which compares the therapeutic effect of PBRT *vs.* photon-based IMRT in the treatment of locally advanced ONB is needed. This study had several limitations. Firstly, this was a retrospective study with a small sample size, and the patients included were from a single institution. Secondly, pathological results of Hyams grade was not collected for all the cases, as it was known that patients with high Hyams grade tumors had a substantially worse survival than those with low Hyams grade tumors.

## Conclusions

In conclusion, our study suggests that frontal lobe invasion is not a poor prognostic factor for patients with ONB. Endoscopic resection in combination with chemo-/radiotherapy is an effective treatment for ONB patients with FLI. However, chemotherapy does not show any benefits in survival, and the role of chemotherapy in the management of ONB patients with FLI needs to be further evaluated.

## Data Availability Statement

The raw data supporting the conclusions of this article will be made available by the authors, without undue reservation.

## Ethics Statement

The studies involving human participants were reviewed and approved by the Eye, Ear, Nose & Throat Hospital of Fudan University. Written informed consent for participation was not required for this study in accordance with the national legislation and the institutional requirements.

## Author Contributions

JW drafted the article and reviewed the submitted version of manuscript. LW worked on the acquisition of data, analysis and interpretation of data. HH critically revised the article and worked on the statistical analysis. YL is responsible for administrative/technical/material support and study supervision. XS worked on conception and design and approved the final version of the manuscript on behalf of all authors. All authors contributed to the article and approved the submitted version.

## Funding

This study was supported by the National Science Foundation of Yunnan Province (No. 2016FA038), Kunming City, China.

## Conflict of Interest

The authors declare that the research was conducted in the absence of any commercial or financial relationships that could be construed as a potential conflict of interest.
