# Supplementary material for: The Treatment Outcomes of Olfactory Neuroblastoma Patients With Frontal Lobe Invasion
Source: Front Oncol. 2021 Jul 5;11:640892. doi: 10.3389/fonc.2021.640892 (PMC8289277; doi:10.3389/fonc.2021.640892)
Supplement: Supplementary file 1 [file DataSheet_1.docx]

**Supplementary Table 1** The chemotherapy mode in the cohort of the 37 patients

| No. | Induction Chemo | Concurrent Chemo | Adjuvant Chemo |
| --- | --- | --- | --- |
| 18 | √ | -- | -- |
| 5 | -- | -- | -- |
| 4 | √ | √ | -- |
| 5 | √ | √ | √ |
| 2 | √ | -- | √ |
| 2 | -- | √ | -- |
| 1 | -- | √ | √ |

Chemo: chemotherapy; No.: number of patients.

| **Supplementary Table 2** Multivariate analysis on OS and PFS of the 37 patients with FLI using the cox proportional hazards model | | | | | |
| --- | --- | --- | --- | --- | --- |
| Variables | 5-year OS | |  | 5-year PFS | |
|  | HR (95% CI) | P value |  | HR (95% CI) | P value |
| Age (>60 vs.<60) | 0.201-5.326 | 0.968 |  | 0.412-8.334 | 0.422 |
| Gender  (Male vs. Female) | 0.081-2.979 | 0.439 |  | 0.085-3.997 | 0.583 |
| Hyams grade  (I/II vs. III/IV) | 0.449-12.792 | 0.306 |  | 0.352-7.459 | 0.551 |
| Cervical LN  (N0 vs. N+) | 1.581-50.502 | 0.013 |  | 0.1471-54.635 | 0.017 |
| Chemotherapy  (No vs. Yes) | 0.069-15.074 | 0.988 |  | 0.153-16.504 | 0.699 |

*Abbreviations:* OS, overall survival; PFS, progression-free survival; FLI, frontal lobe invasion; HR, hazard ratio; CI, confidence interval.
